# Weight prioritized slicing based on constraint logic programming for fault localization

**DOI:** 10.1371/journal.pone.0231331

**Published:** 2020-04-10

**Authors:** Shengbing Ren, Weijia Zhou, Haiwei Zhou, Lei Xia

**Affiliations:** School of Computer Science and Engineering, Central South University, Changsha, China; Shandong University of Science and Technology, CHINA

## Abstract

Fault localization, a technique to fix and ensure the dependability of software, is rapidly becoming infeasible due to the increasing scale and complexity of multilingual programs. Compared to other fault localization techniques, slicing can directly narrow the range of the code which needed checking by abstracting a program into a reduced one by deleting irrelevant parts. Only minority slicing methods take into account the fact that the probability of different statements leading to failure is different. Moreover, no existing prioritized slicing techniques can work on multilingual programs. In this paper, we propose a new technique called weight prioritized slicing(WP-Slicing), an improved static slicing technique based on constraint logic programming, to help the programmer locate the fault quickly and precisely. WP-Slicing first converts the original program into logic facts. Then it extracts dependences from the facts, computes the static backward slice and calculates the statements’ weight. Finally, WP-Slicing provides the slice in a suggested check sequence by weighted-sorting. By comparing it’s slice time and locate effort with three pre-exsiting slicing techniques on five real world C projects, we prove that WP-Slicing can locate fault within less time and effort, which means WP-Slicing is more effectively.

## Introduction

Fault localization, identifying the location of a fault in the program, is a time-consuming and prohibitively expensive task [1]. Nowadays, software usually possesses large-scale and complicated structure which causes state space explosion [2] and makes fault localization difficult. Program slicing is one of the most effective technique for fault localization. The newest research [3] employs a feature selection method to identify those bug-related statements that may cause the program to fail.

Program slicing, since proposed by Weiser in 1984, has been widely used in the field of fault localization, program analysis, symbolic execution and other fields. Existing slicing techniques can be classified into two major types: static slicing and dynamic slicing. Both these two types of techniques can reduce the scale of the statements to be checked in the program by removing irrelevant parts. Static slicing [4] can find all the statements related to the criteria without any external information. Static slice is complete and can satisfy all inputs although its’ size is large. To decrease the slice size, researchers proposed dynamic slicing [5] of many variants [6, 7]. [6] computes the union of dynamic slices for many test cases and [7] consists of integrating dynamic information into the static analysis. [8, 9] combine the dynamic information with the static slicing. [10, 11] compute slices using multi-criteria. These dynamic slicing methods find statements that may affect the criteria under specific input and execution. Sometimes the input or runtime environment is difficult to capture and reproduce. Moreover, some faulty code statements maybe omitted mistakenly. If the input or runtime environment changes, the slice needs to be re-computed. Considering the completeness and versatility of the slice, static slicing is a better choice to be the basic technique. None of the above slicing methods takes into consideration that different statements’ possibilities affecting the criteria (failure point in fault localization) are different. Thus, they spend much effort to check the statements with no priority.

From the research of [12, 13], we can learn that: data dependences are generally stronger than control dependences when propagating effects, such as faults. This theory has been simply applied in [11]. However, a thin slice is computed by directly ignoring control dependences and base pointer flow dependences. Thus, thin slice may miss large quantities of relevant statements and the remaining statements still have no distinction about their possibility. Similarly, [14] proposed prio-slicing also based on this theory. Prio-slicing indicates whether together with how much each statement belongs to a slice. This saves much effort when locating faults. But some flaws still exist: (1) it can only implement on Java-bytecode programs, and (2) in some cases(path between fault and criteria is long or control-dependence dominated), fault has a low probability instead. As a result, its’ effect is even worse than Weiser’s slicing. Moreover, all the above methods can only handle programs in one single language.

As is shown in quantities of applications and our previous researches [15], program transformation has great advantages in the verification or optimization of multi-language programs. An original computer program can be converted into the semantically equivalent constraint logic fact through program transformation. Then constraint logic programming, a form of constraint programming, can conveniently compute the wanted answers according to the logic facts and self-defined constraints [16, 17].

The contributions of this paper are as follows: (1) deriving a weight prioritized static slicing algorithm (WP-Slicing) to help programmers locate faults more quickly and precisely; (2) establishing a mechanism to convert C programs into logic facts in particular form; (3) proposing the constraint rules to extract control and data dependencies from the logic facts; (4) conducting empirical experiments to prove that WP-Slicing is more efficient in most locate tasks.

The rest of this paper is organized as follows. Section ‘Background’ presents the related conceptions for this paper. After giving an illustrative example in Section ‘An illustrative example’, we formalize our approach, WP-Slicing, including the weight formula and algorithm in Section ‘Weight Prioritized Static Slicing’. Section ‘Experimental Setup and Results’ demonstrates the efficiency of our method compared with three pre-existing approaches. Finally, Section ‘Conclusion’ sets out the conclusion and future work of our research.

## Background

### Program slicing

#### Weiser’s slicing

Program slicing [4] was originally proposed by Weiser. By modern standards, Weiser’s slicing is a kind of static backward slicing. Weiser’s slicing computes the set of statements that may affect the slicing criterion (s,v), where s is the specified statement, v is the specified variables set at s, by analyzing the data flow and the control flow information. Weiser’s slicing uses the program dependence graph to store program information and traverses the graph to find all the statements that can reach backwards from the criterion.

#### Thin slicing

In the series of improved methods and variants according to the actual applicative requirements, thin slicing [11] attracts more attention as it can significantly reduce the size of a slice. Thin slicing ignores both control dependences and base pointer flow dependences, only include producer statements for the criteria, which related by producer flow dependences. Thus, a thin slice is tiny, but it excludes many possible faults.

#### Prio-slicing

Different from other methods, prio-slicing [14] distinguishes the visit order of the statements. Prio-slicing computes the probability of reaching each point in program dependence graph from the entry point, the probability of reaching a criterion (expressed by C latter) from a node n in the PDG and the probability of propagating effect (usually manifested as variable change) from node n propagating to C. According to these three probabilities, prio-slicing calculates the likelihood of node n affecting C in execution. The slice statements are sorted by the likelihood from largest to smallest and the sorted result is provided to users as the visit order.

These methods compute slices all by accessing the program dependence graph. Although they can reduce the localization effort, they cannot perform well in the case of multi-language and control-dependence dominated programs.

### Constraint logic programming

Constraint logic programming, the notion of computing with partial information, is becoming recognized as a way of dramatically improving on the current generation of programming languages. More and more researchers attempt to combine it with program slicing. [18] proposed an algorithm named constraint based slicing (Conbas). Conbas reduces the size of dynamic slices using constraint solving. In an empirical evaluation, Conbas reduces the size of dynamic slices by 28% on average for single faults and by 50% for double faults. However, Conbas cannot be applied to large-scale software and can only deal with data of type Integer and Boolean. Also, as a result of developing from dynamic slicing, Conbas cannot avoid the weaknesses of dynamic slicing, such as resulting slice related to the specific input, omitting some relevant statements and so on. Similarly, [17], a method to slice CLP programs, also cannot avoid the weakness of dynamic slicing. Due to the technology foundation of these methods is dynamic slicing, there is no meaning to compare them with our method.

[19] uses constraint analysis concurrently with slicing. The approach applies property-based code slicing to reduce the size of the code to be verified and construct constraint automatically to describe environmental constraints. However, this approach is limited to a specific implementation method. So it can only verify safety properties written in propositional logic.

## An illustrative example

**Fig 1:**
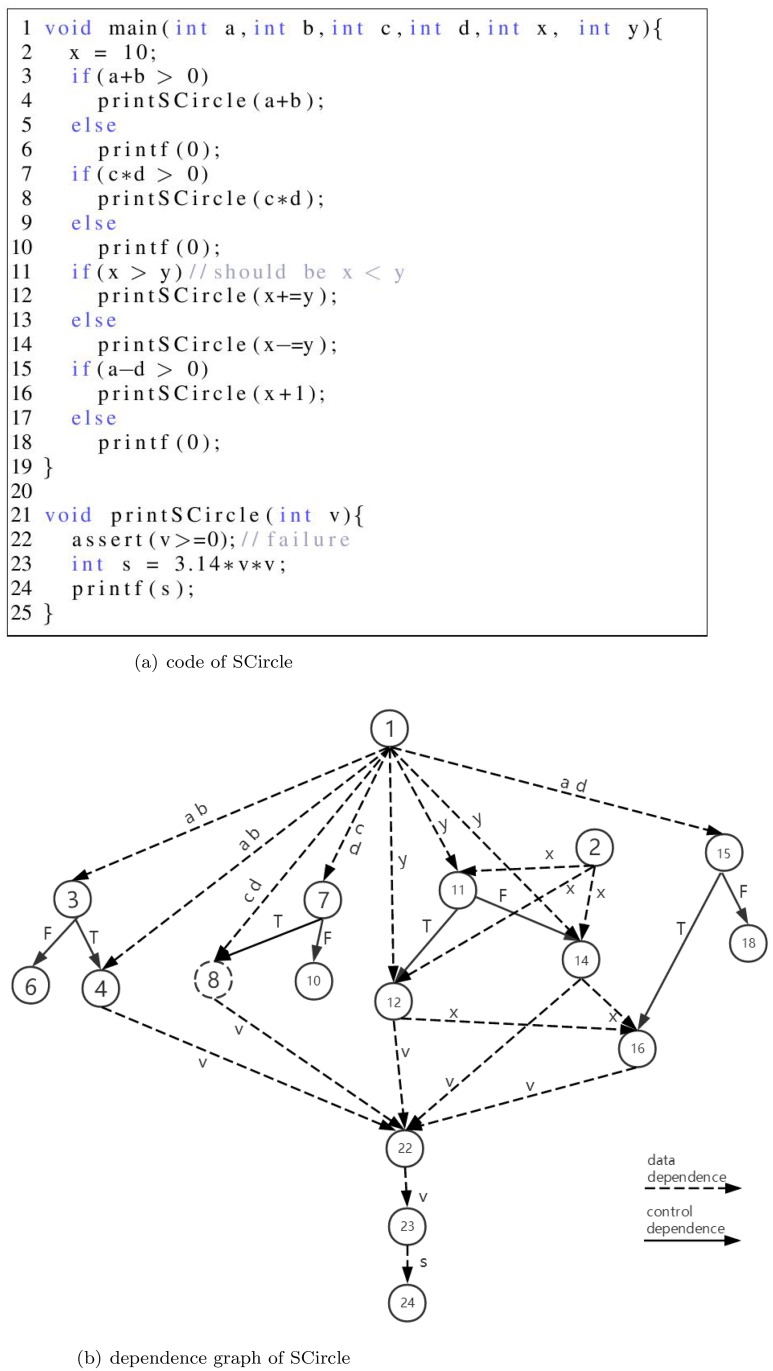
Illustrative program: SCircle.

In this section, we present the distinction among Weiser slice, thin slice, prio-slice and WP-Slice(the slice produced by WP-Slicing), as well as the WP-slicing process using the illustrative program shown in Fig 1.

Suppose that we want to ascertain all statements in this program which may affect the value of v at the statement in line 22. Via using the backward transitive closure of control and data dependences (only producer data dependences in thin slice) from that statement, we obtain the following slices:

Weiser Slice = {1,2,3,4,7,8,11,12,14,15,16,22}, need to check 7 statements to locate fault in line 11.

Thin Slice = {1,2,4,8,12,14,16,22}, fail to find fault.

Prio-Slice = {1:1.0, 2:1.0, 3:0.5, 4:0.5, 7:0.5, 8:0.5, 11:1.0, 12:0.5, 14:0.5, 15:0.5, 16:0.5, 22:1.0}, need to check 4 statements at most to locate fault in line 11.

WP-Slice = {1:0.75, 2:0.75, 3:0.67, 4:0.83, 7:0.67, 8:0.83, 11:0.67, 12:0.83, 14:0.83, 15:0.67, 16:0.83, 22:1.0}, Suggested sequence: {22,2,12,11,15,16,14,4,8,3,1,7} or regenerate the suggested sequence, need to check 4 statements to locate fault in line 11. The method to generate the suggested sequence is explained in the slicing process later.

From the example we can find that both prio-slicing and WP-Slicing distinguish different statements, but the WP-Slicing is more flexible.

**Fig 2:**
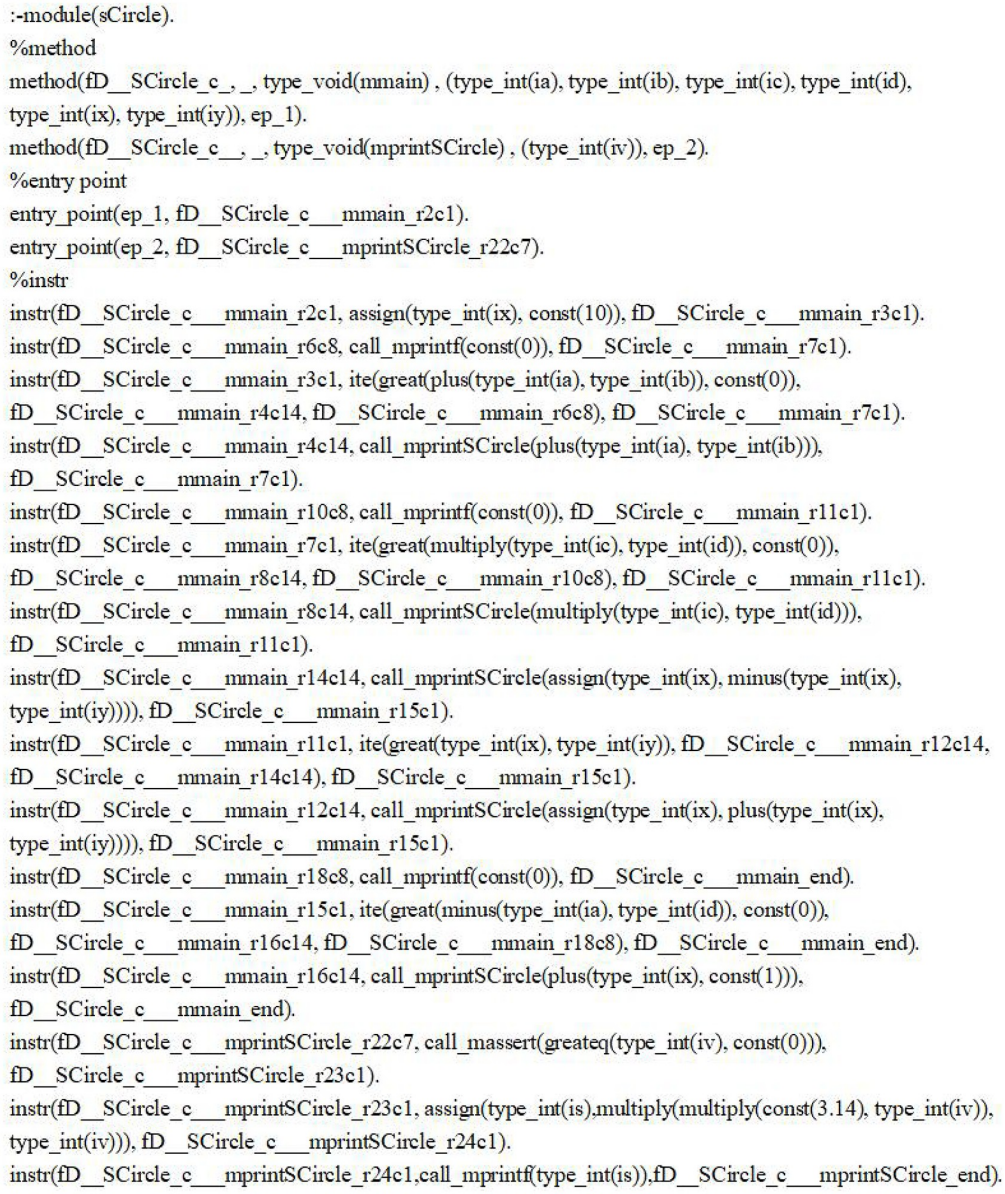
The constraint logic fact of the illustrative program.

The process to compute the WP-Slice of the illustrative program is: first we convert the source code into the constraint logic facts (will be described explicitly in Section ‘Weight Prioritized Static Slicing’) and the constraint logic facts are shown in Fig 2.

Then we extract the dependences from the logical facts, compute slice and calculate the weights of the statements in the slice. Finally, we probabilistic sort the statements in the slice using the weights as the parameter until the user stops the sorting.

## Weight prioritized static slicing

The focus of our work is to design an approach to effectively realize weight prioritized static slicing through program transformation and probability-based selection. We have verified the approach’s feasibility on five programs. The method can also be applied to programs in other languages. Moreover, as we consider the features of multiple languages when designing logical fact structures, multilingual programs can be converted into logical fact. That’s to say, WP-Slicing can deal with multilingual programs in theory. In this section, we present WP-Slicing systematically from program transformation, the constraint rules for extract dependencies fact and the algorithm of WP-Slicing.

### Program transformation and format of facts

At present, we have implemented program transformation on C language, that is, transform files whose suffix is.c into logic facts. Our method uses ANTLR to analyse a.c file’s lexical and syntactic structure and generate a syntax tree. After getting the syntax tree, we access and convert it into corresponding logical fact. We define the definitions of constraint logic facts and depict them in Table 1.

**Table 1:** Some definitions of constraint logic facts.

No.	Source Code	Related Constraint Logic Facts
1	reType methodName(paraList)	*method(meLab,_,reType(methodName), (paraList),epNum). entry_point(epNum,insLab)*.
2	lValue = rValue;	*instr(insLab1,assign(lValue,rValue),insLab2)*.
3	for(declaration;condition;express);	*instr(insLab1,for((declaration;condition;express), forLab),insLab2)*.
4	while(condition);	*instr(insLab1, while(condition,whileLab),insLab2)*.
5	if(condition);	*instr(insLab1,ite(condition,thenLab,elseLab),insLab2)*.
6	switch(var) { case value1: … default: }	*instr(insLab1,switch(var, (value1,case1Lab),…, defaultLab),insLab2)*.
7	methodName(acPara);	*instr(insLab1,call_mmethodName(acPara),insLab2)*.

The first definition in Table 1 is the definition of logic fact for methods, and the remaining ones are the definitions of logic facts for statements in the function, including assignment, for/while loop, etc. The followings are the concrete description:

1. Definition 1 converts a method statement into two facts: method and entry_point. The first element of method fact is the label of the related method, which is composed of the character “f”, the path of the.c file where the method belongs and the.c file’s name. Symbols in a method label like ‘:’ and “∖” is replaced by ‘_’(similarly hereinafter), owing to the nature of the logical program language Prolog. The second element of method fact is a symbol ‘_’. It is used to reserve the location for java class name in future research. The third element stores the return type and name of the method. The forth element is the parameter list for the method and the last element is the id of the entry point for the method.
  The first element of entry_point fact is the id of the entry point and the second is the label of the first statement to be executed in the corresponding method.
2. Definition 2 is the fact for an assignment statement. InsLab1 is composed of the label of the belonged method, the belonged method name, character ‘r’, the row number for this statement, character ‘c’, the column number for this statement(All statements’ labels are composed like this). Element ‘lValue’ means the left value of assignment and ‘rValue’ means the right value. InsLab2 is the label of next statement.
3. Definitions 3 and 4 are the fact for loop statements, which are the ‘for’ and the ‘while’. The fact of ‘for’ statement is definition 3. The first element ‘insLab1’ is the label of ‘for’ statement.
  The second element ‘for((declaration;condition;express),forlab)’ contains the expressions of for statement in the innermost parentheses and the label of the first statement to execute in the loop(’forlab’). If not entering the loop, the process will execute the statement corresponding to insLab2.
  The fact of ‘while’ statement is similar to the ‘for’ statement’s fact.
4. Definitions 5 and 6 are the facts for judgement statements: if-else and switch-case. Definitions 5 is the fact of if-else struct. The first element ‘insLab1’ is the label of ‘if’ statement. The elements in the second part: ‘condition’ is the condition expression in ‘if’ statement; ‘thenLab’ is the label of the first statement if the condition expression is satisfied; ‘elseLab’ is the label of the first statement if the condition expression is not satisfied. Meanwhile, if there are no statements for else, ‘elseLab’ is the same as ‘insLab2’—the label of the first statement after the if-else selection structure.
  Definition 6 is the fact of switch-case struct whose first element ‘insLab1’ is the label of ‘switch’ statement. The remaining elements represent the object in ‘switch’(may be a variable or expression, etc), the value of first case and it’s first statement’s label, the couple of other cases’ values and labels(if have), the label of the first statement if all the cases cannot meet, the first statement to be executed after the switch-case structure.
5. Definition 7 is the fact of a statement of invoking a function. The element ‘call_mmethodName(acPara)’ stores the invoked method’s name and the parameters actually passed.

### Constraint rules for extract dependencies fact

We present the dependencies extraction rules in Table 2. Rules 1-7 are the rules for control dependencies extracting and some other facts needed to compute data dependencies. Rules 8-9 are the rules for data dependencies extracting. We use a label to refer to the corresponding statement.

**Table 2:** Rules to extract control and data dependences from facts.

Rule No.	Rules to Extract Dependences
1	*control*(*cdLab,lab*1) ⋀ *endControl*(*cdLab,endLab*) ⋀ *lab*2 < *endLab* ⇒ *control*(*cdLab,lab*2)
2	*instr*(*lab*1, *for*((*declaration*; *condition*; *express*), *forLab*), *Lab*2) ⇒ *control*(*lab*1, *forLab*) ⋀ *endcontrol*(*lab*1, *lab*2) ⋀ *flow*(*lab*1, *forLab*) ⋀ *flow*(*lab*1, *lab*2) ⋀ *defF*(*lab*1, *dV*) ⋀ *refF*(*lab*1, *rV*)
3	*instr*(*lab*1,*while*(*condition*, *whileLab*), *lab2*) ⇒ *control*(*lab*1, *whileLab*) ⋀ *endcontrol*(*lab*1, *lab*2) ⋀ *flow*(*lab*1, *whileLab*) ⋀ *flow*(*lab*1, *lab*2) ⋀ *refF*(*lab*1, *rV*)
4	*instr*(*lab*1, *ite*(*condition*, *thenLab*, *elseLab*), *lab*2) ⋀ *elseLab* ≠ lab2 ⇒ *control*(*lab*1, *thenLab*) ⋀ *control*(*lab*1, *elseLab*) ⋀ *endcontrol*(*lab*1, *lab*2) ⋀ *flow*(*lab*1, *thenLab*) ⋀ *flow*(*lab*1, *elseLab*) ⋀ *refF*(*lab*1, *rV*) *instr*(*lab*1, *ite*(*condition*, *thenLab*, *elseLab*), *lab*2) ⋀ *elseLab* = *lab*2 ⇒*control*(*lab*1, *thenLab*) ⋀ *endcontrol*(*lab*1, *lab*2) ⋀ *flow*(*lab*1, *thenLab*) ⋀ *flow*(*lab*1, *lab*2) ⋀ *refF*(*lab*1, *rV*)
5	*instr*(*lab*1, *switch*(*var*, (*value*1, *case*1*Lab*), …, *defaultLab*), *lab*2) ⇒*control*(*lab*1, *case*1*Lab*) ⋀ … ⋀ *control*(*lab*1, *defaultLab*) ⋀ *endcontrol*(*lab*1, *lab*2) ⋀ *flow*(*lab*1, *case*1*Lab*) ⋀ … ⋀ *flow*(*lab*1, *defaultLab*) ⋀ *refF*(*lab*1, *rV*)
6	*instr*(*lab*1, *assign*(*lValue*, *rValue*), *lab*2) ⇒*computeControl*(*lab*1, *lab*2) ⋀ *flow*(*lab*1, *lab*2) ⋀ *defF*(*lab*1, *dV*) ⋀ *refF*(*lab*1, *rV*) *instr*(*lab*1, *assign*(*lValue*, *rValue*), *lab*2) ⋀ *find* ‘call_m’ *in* *rValue* ⇒*computeControl*(*lab*1, *cmLab*) ⋀ *flow*(*lab*1, *cmLab*) ⋀ *defF*(*lab*1, *para*)
7	*instr*(*lab*1, *call_mmethodName*(*para*), *lab*2) ⇒ *computeControl*(*lab*1, *cmLab*) ⋀ *computeControl*(*lab*1, *lab*2) ⋀ *flow*(*lab*1, *cmLab*) ⋀ *flow*(*lab*1, *lab*2) ⋀ *defF*(*lab*1, *para*) ⋀ *refF*(*lab*1, *rV*)
8	*flow*(*X*, *Y*)⋀¬*defF*(*X*, *V*)⇒*purepath*(*X*, *Y*, *V*) *flow*(*X*, *T*)⋀¬*defF*(*X*, *V*)⋀*purepath*(*T*, *Y*, *V*)⇒*purepath*(*X*, *Y*, *V*)
9	*defF*(*X*, *V*)⋀*refF*(*Y*, *V*)⋀*flow*(*X*, *Y*)⇒*dataDep*(*X*, *Y*, *V*) *defF*(*X*, *V*)⋀*refF*(*Y*, *V*)⋀*flow*(*X*, *T*)⋀*purepath*(*T*, *Y*, *V*)⇒*dataDep*(*X*, *Y*, *V*)

Rule 1 means that if lab1 control depends on cdLab and lab2 is not out of the control range, then lab2 is also control-dependent on cdLab. The ‘lab2<endLab’ refers to three conditions: (1)lab2, endLab are in the same method and lab2’s row number is smaller than endLab’s; (2)lab2, endLab are in the same method, their row numbers are the same and lab2’s column number is smaller than endLab’s; (3)lab2 and endLab are in different methods. If meeting any one of the conditions, lab2 is in the control range of cdLab. This rule will be used later.

Rules 2, 3 are the extraction rules for loop statements. If the instr is a ‘for’ one, then forLab control depends on lab1 and this control range ends at lab2. Moreover, there is a flow from lab1 to forLab(lab2), that to say, the next instr to be executed after lab1 is forLab(lab2). Some variables are defined or referenced at the ‘declaration’, ‘condition’ and ‘express’ of lab1. Normally, ‘declaration’ and ‘express’ are assignment expressions and ‘condition’ is a judgement expression. Variables ‘dV’ are the variables in the left value of ‘declaration’ and ‘express’, and variables ‘rV’ are the variables in ‘condition’ and right value of ‘declaration’ and ‘express’. Rule 3 is similar to rule 2. The only difference is that there are only variable references in the condition of lab1.

Rules 4, 5 are the extraction rules for judgement statements. If the instr is a ‘ite’ one and its’ elseLab is not equal to lab2, then thenLab and elseLab control depend on lab1. Control range ends at lab2. Flow from lab1 to thenLab, from lab1 to elseLab and variable references in the ‘condition’ of lab1 also exist. If the instr is a ‘ite’ one but its’ elseLab is equal to lab2, only thenLab depends on lab2. Flow and refF facts are the same. Similarly in rule 5 for ‘switch’ statements.

Rule 6 is the extraction rule for assignment statements. First, computing the control dependency of lab2 through lab1 using rule 1. Also, there is a flow from lab1 to lab2. The dV refers to the variables defined or redefined in lValue, and rV refers to the variables referenced in rValue. Meanwhile, if there is a user-defined function call in rValue, control dependency of cmLab(entry lab of called function) should also be computed using rule 1. There is also a flow from lab1 to cmLab. Variables ‘para’ are formal parameters of the called function.

Rule 7 is the extraction rule for function call statements. Control dependency of cmLab, as well as lab2, should also be computed using rule 1. There are flows from lab1 to cmLab, from lab1 to lab2. Variables ‘para’ are formal parameters of the called function and variables ‘rV’ are the actual parameters.

With the ‘flow’, ‘defF’ and ‘refF’ facts, we can understand rules 7, 8. If there is a flow from X to Y and no define on V at X, we find the fact ‘purepath(X,Y,V).’ (i.e. no redefine on V from X to Y). More purepath can be found iteratively. Using the purepath defined in rule 7, rule 8 extracts data dependencies. Y is data-dependent on X when V is defined at X and referenced at Y and there is a flow from X to Y. Or when there exists a purepath of V from X to Y after V defined at X and V should be referenced at instr Y. Only in these two cases, Y is data-dependent on X.

All of these extraction rules can be reused. Moreover, we can get dependencies with less space and time which avoids state space explosion since we use no graph.

### Algorithm of WP-Slicing

Algorithm 1 shows the WP-Slicing algorithm that computes the WP-Slice from the original program. First, the algorithm converts the original program into constraint facts using ANTLR and extracts both control and data dependencies. After getting the dependencies fact, the algorithm queries the dependencies fact backwards from a slicing criterion using a breadth-first strategy.

**Algorithm 1** WP-Slicing algorithm

**Input** *P*: program; *C*: slice criterion; *resort*: resort flag

**Output** *S*: WP-Slice; *Q*: suggested sequence

1: constraint facts set *F* = convert(*P*);

2: *control*(*X*, *Y*) = computeControlDepFacts(*F*);

3: *dataDep*(*X*, *Y*, *V*) = computeDataDepFacts(*F*);

4: *weightQ* = {*C*};

5: *C*.*depth* = 0;

6: *S* = {*C*: 1.0};

7: *depPre* = {};

8: *dataDepPre* = {};

9: *resort* = *true*

10: **repeat**

11:  *n* = *weightQ*.*pop*();

12:  if *control*(*X*, *n*) has solution:

   *depPre*.append(*X*.*value*);

13:  if *dataDepF*(*X*, *n*, *V*) has solution:

   *dataDepPre*.append(*X*.*value*);

   *depPre*.append(*X*.*value*)

14:  **repeat**

15:   *m* = *depPre*.*pop*();

16:   if *m* ∈ *weightQ*: goto 11;

17:   *m*.*depth* = *n*.*depth* − 1;

18:   if *m* ∈ *dataDepPre*:

    *S*.*append*(*m*: *S*[*n*] − 2^*m*.*depth*^/3)

     else: *S*.*append*(*m*: *S*[*n*] − 2^*m*.*depth*^/3*2)

19:   *weightQ*.*append*(*m*)

20:  **until** *depPre* is *ϕ*;

21: **until** *weightQ* is *ϕ*

22: **repeat**

23:  *Q* = rouletteWheelSelection(*S*)

24:  read(*resort*)

25: **until** *resort* is *false*

26: return *S*, *Q*

The method used to compute an instr’s weight can be summarized as:

$$\begin{array}{c} \begin{array}{c} m.depth=n.depth-1\\ m.weight=\{\begin{array}{ll} n.weight-1/3*2^{m.depth}, & if\;dataDep(m,n).\\ n.weight-2/3*2^{m.depth}, & if\;control(m,n). \end{array} \end{array} \end{array}$$ (1)

In Eq 1, the weight of a control dependence predecessor’s declines more than a data one. By this means, we simply distinguish the data dependencies and control dependencies. After getting the weight of each instr, we use it as a parameter to probabilistic sort instrs. Instrs with high weights are more likely to be in the front rank. This is what ‘weight prioritized’ mean. Using this sorting mechanism, instrs with high weights have more possibility to be checked early, while instrs with low weights also have the possiblity (though less than instrs with high weights) to be cheacked early. Comparing to the highest-first strategy, this balance two situations: the path between fault and failure is data-dependence dominated and control-dependence dominated.

The function convert(*P*) in step 1 transforms source code into constraint logic facts. It returns the semantically equivalent constraint logic facts. Function computeControlDepFacts(*F*) in step 2 and computeDataDepFacts(*F*) in step 3 extract and return control dependence facts and data dependence facts from the program fact F. Steps 4-9 initialize the data structures for the intermediate calculation and for the output, which is the weight-map *S*, by seeding it with the slicing criterion and weight 1.0. We use *weightQ* to store instrs when computing slice, *depPre* to store the dependence predecessors of current instr, *dataDepPre* to store the data dependence predecessors of current instr.

The first loop in steps 10-21 proceeds until no new nodes are left to process in the weight queue until the dependence facts have been completely queried. Step 11 picks the next instr n to query, which is the node with the highest weight, and deletes it from the weight queue. Steps 12-13 query and store the dependence predecessors of n. Steps 13-19 compute the predecessors’ weight and append them to *S* together with their weights. Also, the dependencies of n are added to weightQ for the outer loop. Steps 22-25 probabilistic order the instrs in slice using roulette algorithm until the user’s input for *resort* is *false* in step 24.

## Experimental setup and results

Our empirical studies are designed to answer the following research questions:

**RQ1**: How practical is WP-Slicing in terms of time cost?

**RO2**: How effective is WP-Slicing for fault localization compared to Weiser slicing, thin slicing and prio-slicing?

We perform all our experiments on a computer with configurations: core i7 CPU, 8G RAM, 64-bit Windows 10.

### Setup

The five projects used in our empirical studies are all real-world projects: schedule and printtokens are files from Siemens suite(http://sir.csc.ncsu.edu/portal/index.php), unreach_call and incomplete are files from the International Competition on Software Verification SV-COMP(https://github.com/sosy-lab/sv-benchmarks), and FFmpeg(https://github.com/FFmpeg/FFmpeg) is a collection of libraries and tools to process multimedia content on Github. For the third and forth programs, we just take a part of their original name to refer them, as the original names really too long. We present the detailed information and some experiment settings of these programs in Table 3, including lines of code(LOC), numbers of facts converted(NOF)(with some necessary comments), number of faults.

**Table 3:** Subject programs detail and faults settings.

Program	LOC	NOF	Faults
schedule	410	145	2
printtokens	563	241	3
unreach	595	516	3
incomplete	3514	1553	5
FFmpeg	49359	39681	10

Table 3 shows that NOF of a subject program is usually less than it’s LOC. There are mainly three reasons: a. usually there is only one statement in a line; b. some statements, such as comments, do not need to convert into logical fact; c. some statemnts, such as declarations, can be revealed together with other statements. We respectively set 2, 3, 3, 5, 10 faults for these five programs.

For each program’s faults, our experiment follows the following steps:

1. Identify the failure: We identify the first statement that deviates from the correct service behavior due to the fault, which usually appears to either print the first wrong value or throw an uncaught exception, etc. This statement would be used as the slice criterion later on.
2. Preprocess the source code: Different from Weiser slicing and other techniques’ preprocessing(obtain the static dependence graph), WP-Slicing converted the source code into the constraint facts as described in the first part of section ‘Weight Prioritized Static Slicing’.
3. Apply each technique: We applied each technique(WP-Slicing, Weiser slicing, thin slicing, prio slicing), recorded their running time and computed the effort for localizing the fault, which is defined as:
  $$\begin{array}{c} effort=\frac{checked(f)}{programsize} \end{array}$$ (2)
  The parameter *checked*(*f*) is the number of the statements needed to be checked before fault *f* found, and *programsize* is the total number of statements in the program. We used breadth-first backward travesals of the graph for Weiser slicing, thin slicing and prio-slicing.

### Results

Table 4 shows the average running times(seconds) and standard deviation for each technique per subject with different faults and for all faults. The average slice time shows that WP-Slice takes a bit more time than weiser slices and thin slices, but much less than prio-slice. Comparing the standard deviation, we observe that WP-Slice time is relatively stable. This is because it needs to first analyze logical facts and extract control dependencies and data dependencies, which are done in source code preprocessing stage in other methods(with extensive computations).

**Table 4:** Statistics for slice time of WP-Slice, weiser’s slice, thin slice and prio-slice in seconds.

Program		WP-Slice	Weiser’s Slice	thin Slice	prio-Slice
schedule	**avg**	**1.57**	**1.51**	**1.64**	**1.74**
	st.dev	0.0283	0.0566	0.0849	0.0424
printtokens	**avg**	**1.94**	**1.63**	**1.84**	**2.16**
	st.dev	0.2381	0.0569	0.0586	0.2996
unreach	**avg**	**2.01**	**1.83**	**2.07**	**2.15**
	st.dev	0.1249	0.0625	0.0757	0.2994
incomplete	**avg**	**12.17**	**10.09**	**12.24**	**13.11**
	st.dev	1.3159	1.0383	1.1889	2.1882
FFmpeg	**avg**	**548.72**	**394.61**	**402.45**	**2768.74**
	st.dev	35.5528	43.9509	37.4032	93.8627
all faults	**avg**	**241.87**	**174.35**	**178.29**	**1207.36**
	st.dev	276.1375	199.5433	202.4778	1401.4873

WP-Slice calculates the slice using query mechanism, together with the calculation of the weight for each statement, which is much faster than access dependence graph. The weight calculation method we used is simple and ensures different dependences have different importance, as our check sequence is not absolutely dependent on the weight. Both of these save much running time than prio-slicing. Thus, for large scale programs, such as FFmpeg, WP-Slice is superior in running time. For per 1000 lines code, WP-Slice uses 38.7% of execution time more than Weiser’s slice((time of WP-Slice—time of Weiser’s slice)/time of Weiser’s slice, similarly hereinafter), while thin slice uses 2.3% more and prio-slice uses 592.5% more than Weiser’s slicing. Thus WP-Slicing is practical in terms of time cost.

**Fig 3:**
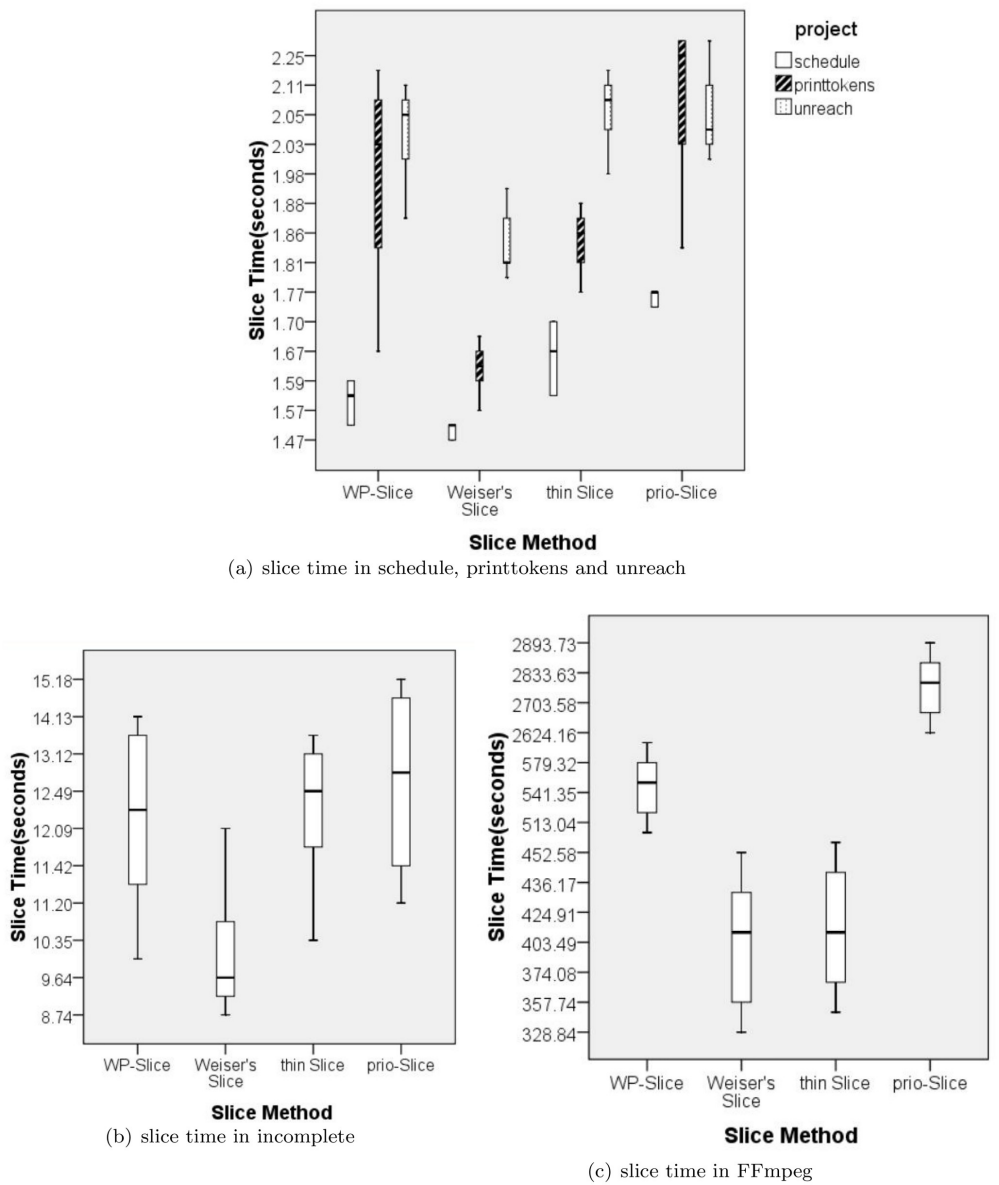
Box-plot for slice time of all slice methods on different projects.

Fig 3 is the box-plots for slice time of four slice methods on these five real-world projects. Considering that the slice time of schedule, printtokens and unreach is approximate and the data volume is not too much, we combined the slice time of the first three items into Fig 3(a). From the distributions shown in Fig 3(a)–3(c), we can observe that: When the project’s sclae is not too large, the slice times of these four methods are relatively close. The specific order is: Weiser’s slice is the least one and prio-slice is the most one. Weiser’s slice and thin slice are in the middle and their relationship of size is related to the project’s complexity and scale. When the project’s sclae is large, Weiser’s slice time is the least,thin slice uses slightly more. WP-Slice uses a little more time to slice than Weiser’s slice and thin slice but much less than prio-slice. This is because WP-Slice needs to convert source code to logic fact first and compute weights for statements in slice. Dueing to the simpleness of weight calculation method, WP-Slice uses about a quarter of prio-slice time as prio-slice’s method to compute probability is really complex.

Table 5 shows the result of the size of the sliced-fact or slice and the effectiveness comparisons among these four techniques.

**Table 5:** Comparison of slice fact(*μ*_*fact*_) or slice size(*μ*_*slice*_) and localization effort(*ν*_*effort*_) among WP-Slice, weiser’s slice, thin slice and prio-slice.

	fault	WP-Slice		Weiser’s Slice		Thin Slice		Prio-Slice	
		*μ*_*fact*_	*ν*_*effort*_	*μ*_*slice*_	*ν*_*effort*_	*μ*_*slice*_	*ν*_*effort*_	*μ*_*slice*_	*ν*_*effort*_
schedule	f1	48	6.59%	48	9.27%	35	**5.61%**	48	7.32%
	f2	73	**4.63%**	73	13.17%	26	fail	73	14.39%
printtokens	f1	52	**3.73%**	52	6.93%	31	4.09%	52	6.04%
	f2	85	**7.28%**	85	14.03%	46	fail	85	9.41%
	f3	119	**6.75%**	119	14.74%	37	fail	119	16.34%
unreach	f1	77	5.21%	77	9.08%	53	fail	77	**4.42%**
	f2	61	**6.72%**	61	7.23%	48	fail	61	10.25%
	f3	103	**7.56%**	103	8.91%	57	fail	103	9.41%
incomplete	f1	613	**12.15**%	613	15.20%	497	fail	613	13.86%
	f2	572	11.13%	572	12.89%	425	**10.07%**	572	14.40%
	f3	748	14.68%	748	18.73%	542	fail	748	**13.94%**
	f4	473	5.61%	473	8.39%	368	**4.95%**	473	7.85%
	f5	961	**17.87%**	961	24.39%	658	fail	961	21.12%
FFmpeg	f1	16743	17.35%	16743	25.42%	9887	fail	16743	**12.23%**
	f2	8259	**15.48%**	8259	16.05%	5983	fail	8259	16.54%
	f3	20135	**21.12%**	20135	37.51%	10678	fail	20135	22.13%
	f4	25917	18.67%	25917	26.85%	11329	fail	25917	**17.96%**
	f5	18782	**13.20%**	18782	27.26%	7641	14.44%	18782	16.64%
	f6	21896	13.01%	21896	19.23%	9729	fail	21896	**10.50%**
	f7	6164	**10.11%**	6164	10.65%	4692	fail	6164	12.58%
	f8	26707	**31.66%**	26707	35.59%	7842	fail	26707	38.93%
	f9	18439	1.54%	18439	1.94%	5887	1.82%	18439	**1.45%**
	f10	13767	**1.99%**	13767	2.75%	6931	fail	13767	10.20%

We compute the effort needed to localize fault using Eq 2. The most effective effort is marked in bold. The ‘fail’ means that thin slice cannot find the fault. This usually appears when there exist control dependencies or base pointer flow data dependencies between fault and failure since thin slice ignores them. The results in Table 5 indicate that in most cases, WP-Slice is the optimal solution. Even when WP-Slice is not the optimal ones, the disparity between WP-Slice and the optimal ones is not too much. Since we assign different weights to different statements and use the weight as the parameter to do roulette wheel selection sorting, a statement with bigger weight(more probability to propagate faults) has more probability to on the front row in checked sequence. Meanwhile, if the weight of the fault is low, it also has a probability to be checked earlier(often earlier than in prio-slicing). When the path between fault and failure is control-dependence dominated(such as f2 in schedule) or the distance between them is long(such as f8 in FFmpeg), WP-Slicing localizes the fault more effectively and performs better than Weiser’s slicing, thin slicing as well as prio-slicing. When the path is data-dependence dominated and the distance is not long (the case prio-slicing expert at), WP-Slicing also has the probability to perform slightly better than prio-slicing, at least better than Weiser’s slices. Therefore, WP-Slicing is more effective than others on the whole.

## Conclusion

Program slicing is an effective means when programmers need to find faults because it can delete irrelevant code scope to reduce the scale of possible fault statements. However, the slice produced is often too large and without focus to localize faults rapidly.

In this paper, we propose a new static slicing technique called WP-Slicing, which distinguish and sort different statements using weight. In an empirical evaluation, we show that WP-Slicing can localize faults more effectively than Weiser’s slicing. In some cases, it performs better even than thin slicing and prio-slicing. Especially when the path between fault and failure is long or control-dependence dominated in which situation thin slicing can not work and prio-slicing is inefficient. In the majority of cases, WP-Slicing works best, and second-best in a few cases. WP-Slicing is never the worst one. Thus, WP-Slicing should be the preferred method for programs existing lots of control dependencies or when the dependencies of the program are not clear. This method can also be applied for multi-language programs.

We intend to introduce some dynamic informations for weight calculation. In future work, We plan to make the weight more accurate to reflect the probability of a statement to be the fault without extra computation time.
